# Boosting the Transparency of Thin Layers by Coatings of Opposing Susceptibility: How Metals Help See Through Dielectrics

**DOI:** 10.1038/srep20659

**Published:** 2016-02-10

**Authors:** Mohammed Al Shakhs, Lucian Augusto, Loïc Markley, Kenneth J. Chau

**Affiliations:** 1 School of Engineering, The University of British Columbia, Kelowna, British Columbia, Canada

## Abstract

We propose a hypothesis that a very thin layer can be made more transparent by adding a thin coating with susceptibility of opposing sign. Two experimental tests backed by a theoretical model support this hypothesis. First, we show that the visible and near-infrared transmission through a semi-transparent silver film can be enhanced by up to ~70% and spectrally tailored depending on the type and thickness of the dielectric coating. Material types explored as dielectric coating layers include conventional metal oxides (titanium dioxide) and lesser-explored elemental semiconductors (undoped silicon, p-type silicon, and germanium). Second, and more surprisingly, we show that coating a 50-nm-thick silicon nitride membrane with a 10-nm-thick silver layer can modestly enhance the transmission by up to 6 ± 1% in the blue part of the spectrum. Transmission enhancements are observed for three silver-coated membranes in different configurations. Thinner silver coatings are theoretically capable of enhancement factors greater than 10%, but implementation is restricted by challenges in making smooth and continuous silver films below 10 nm in thickness. This study is important because it is the first demonstration of reciprocity with respect to the transmission enhancements achieved by combining thin metallic and dielectric layers.

It has been known since the 1950s that the transparency of low-loss metallic films can be boosted by coating with a thin, high-index dielectric layer^1^,^2^,^3^,^4^,^5^. This phenomenon has led to important developments over a range of energy and information technologies. Dielectric-coated metal layers are the critical component of energy-efficient heat-reflecting windows^6^,^7^,^8^,^9^,^10^ and show promise as cheap and flexible transparent conductors^11^,^12^,^13^,^14^ that could supplant indium tin oxide for mainstream integration in photovoltaics and displays. Recently, the combination of thin metal and dielectric layers has been explored to build new nanophotonic devices, such as spectrally selective transmission filters^15^,^16^,^17^,^18^, flat lenses^19^,^20^,^21^,^22^,^23^, and new types of materials with unique effective parameters^24^,^25^,^26^. Even in these recent applications, the incorporation of dielectric layers generally serves the purpose of boosting the transparency of metallic layers that would otherwise be opaque. Here we show that transmission enhancements realized by combining metallic and dielectric layers are reciprocal. In particular, a thin dielectric layer can be made more transparent over parts of the visible spectrum by the addition of a thin metallic layer.

We derive a general condition based on Maxwell’s equations showing that a very thin layer composed of either dielectric or metal can be made more transparent by coating with a material having a susceptibility of opposing sign. This is corroborated by experimental measurements of light transmission through dielectric-coated silver films and silver-coated silicon nitride membranes, which compare favorably to calculations. Coating silver films with thin layers of titanium dioxide, undoped silicon, boron-doped p-type silicon, and germanium is shown to yield large transmission boosts up to about 70%, which are spectrally tunable over the visible and near-infrared by variations in the material type and layer thickness. Coating a 50-nm-thick silicon nitride membrane with sub-10-nm-thick layers of silver is shown to yield transmission boosts up to 6 ± 1%, albeit restricted to the blue part of the visible spectrum. Silver coatings thinner than 10 nm are theoretically capable of inducing visible-band transmission enhancements greater than 10%, but experimental realization of such enhancement factors is restricted by difficulties in creating sub-10-nm-thick silver layers that are continuous and planar.

## Theory

**Figure 1:**
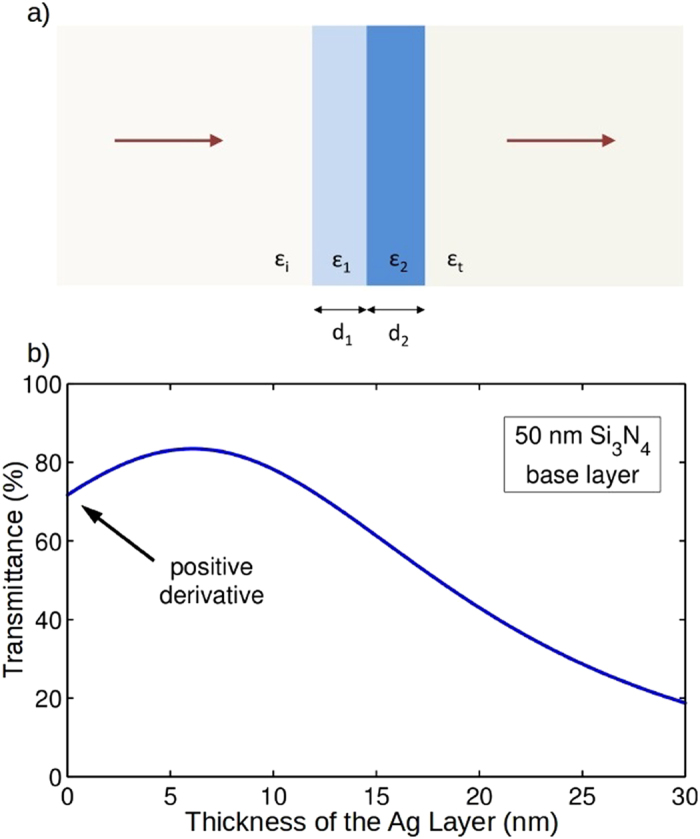
(**a**) Ideal configuration of a bi-layer immersed in two half-spaces, illuminated at normal incidence from the left half-space. (**b**) Predicted normal-incidence transmittance at a wavelength of 650 nm through a bilayer composed of a 50-nm-thick base layer of silicon nitride and a coating layer of silver of variable thickness. A positive derivative of the transmittance in the limit of zero coating layer thickness can be used as an indicator of transmission enhancement.

We begin by deriving an expression to show that transmission enhancements conferred by a coating layer to a base layer can occur when the layer susceptibilities have opposite sign. Consider the configuration in Fig. 1(a) depicting a bi-layer immersed in two semi-infinite half-spaces. The first layer is the base layer of thickness 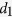 and complex permittivity 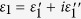 (with corresponding complex refractive index 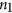 and complex susceptibility 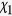), and the second layer is the coating layer of thickness 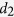 and complex permittivity 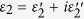 (with corresponding complex refractive index 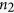 and complex susceptibility 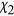). The transmittance, *T*, of a monochromatic plane wave at frequency *ω* normally incident from the left half-space onto the bilayer can be solved using standard transfer matrix methods^27^ and has the general functional form


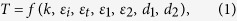


where the left and right half-spaces have respective real permittivity values of 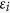 and 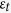, *k* = *ω*/*c* is the free-space wave vector, and *c* is the speed of light.

A simplified condition for transmission enhancement in the thin-film limit can be derived from the derivative of the transmittance with respect to 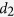 in the limit where 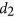 goes to zero. If we assume high figures of merit for the base and coating layers (ie., complex permittivities are predominantly real such that 
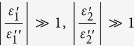
), the condition to achieve transmission enhancement is given by


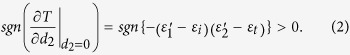


In the case of a bi-layer immersed in air (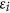 = 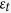 = 1), the condition for transmission enhancement simplifies to 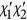 <0, where 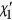 and 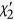 are the respective real susceptibilities of the base and coating layers. Thin bi-layers with opposing susceptibilities are more transparent than the individual layers alone, independent of the layer ordering. The optimal coating layer thickness to achieve maximal transmission satisfies the following


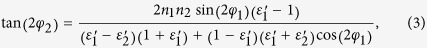


where 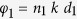 and 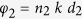. Equation 3 is valid for all permittivities that satisfy (2) and layers of any thickness.

We will consider transmission enhancements when the permittivity values of the base and coating layers satisfy either


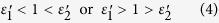


The first condition in (4) can be realized in the well-studied configuration of a metallic layer coated with a dielectric layer. The second condition in (4), which has yet to be examined, can be realized in the configuration of a dielectric layer coated with a metallic layer.

The prediction of transmission enhancement conferred by a thin metallic layer is counter-intuitive but consistent with solutions to Maxwell’s equations describing the transmission through planar layered media. For example, Fig. 1(b) illustrates the calculated normal-incidence transmittance at a wavelength of 650 nm of a 50-nm-thick silicon nitride base layer as a function of the thickness of a silver coating layer, which show a maximal transmission boost of about 10% when the silver layer is 6.1 nm thick. The calculations are made assuming complex optical constants of silver and silicon nitride from ref. 28 and ref. 29, respectively. The optimal silver layer thickness extracted from Fig. 1(b) matches well with the optimal thickness 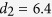 nm calculated from (3), which assumes negligible losses in silver.

Enhanced transmission arises from effective optical path length reduction. The optical path length of a layer of negative susceptibility (NS) is smaller than an equally thick layer of free space. When the NS layer is paired with a layer of positive susceptibility (PS), the optical path length through the bi-layer is reduced relative to that of the PS layer alone. If the thickness of the PS layer is less than a quarter wavelength, reduction in the optical path length results in an increase in transmission. Furthermore, if the NS layer is metallic, its thickness must be sufficiently thin so that transmission reduction due to reflection and attenuation is less than transmission enhancement due to optical path length reduction.

## Methodology

To investigate transmission enhancements due to combinations of metallic and dielectric layers, thin-film samples composed of metal (99.99% Ag), metal-oxide (99.9% TiO_2_), and elemental semiconductors (99.999% Si, 99.999% p-Si, 99.999% Ge) are prepared by magnetron sputter deposition (Angstrom Engineering Nexdep). These materials are chosen based on their properties in the visible region: silver has a real, negative susceptibility and the highest figure of merit amongst common metals; TiO_2_ has a real, positive susceptibility, high figure of merit, and high chemical stability; Si and Ge have real, positive susceptibilities and modest figures of merit, are compatible with physical vapor deposition, and can be tuned by impurity doping. After the deposition chamber reaches a base vacuum pressure of at least ~5 × 10^−5^ torr, the films are sputtered at room temperature and an argon gas pressure of 3 × 10^−3^ torr. The deposition rates of the dielectric and silver films are set to 1 Å/s and 2 Å/s, respectively. Substrates are mounted on a rotating platform spaced about 20 cm above 5-cm-diameter targets. Two families of samples are made to test transmission enhancement conferred by either dielectric or metallic coating layers.

For one family of samples, the base layer is made by sputtering silver onto borosilicate glass substrates and the coating layer is made by *in situ* sputter-coating the silver with either TiO_2_, Si, p-Si, or Ge. For each type of coating layer, five samples are made in which the silver base layer thickness is fixed while the dielectric coating layer thickness varies from 18 nm to 60 nm. Silver films coated with TiO_2_ are a well-established recipe for realizing transparent conductors^6^ and, in this study, serve as benchmarks. Transmission enhancements for silver films coated with semiconductors like Si or Ge have yet to be explored, although related studies have shown that optically-thick metallic substrates coated with ultrathin layers of Si and Ge can produce a wide range of reflected colors^30^,^31^,^32^,^33^.

For the other family of samples, the base layer is a free-standing 50-nm-thick, 0.5 mm ×0.5 mm silicon nitride membrane (SPI Supplies) and the coating layer is made by sputter-coating the membrane with silver. Based on the manufacturer’s specifications, thickness variation between different membrane samples is less than 5 nm and the surface roughness is better than 0.5 nm root mean squared. The free-standing silicon nitride membrane is used because transmission enhancements of a metal-coated dielectric layer are predicted to be most pronounced when the bounding media is air. In some cases, the silver coating layer is passivated *in situ* by an additional layer of sputtered TiO_2_ to prevent atmospheric corrosion.

The thicknesses of the deposited films are measured *in situ* by a quartz crystal monitor, which was calibrated by depositing a series of thin-film samples of variable thickness for each type of material and measuring their thicknesses with a stylus-based profilometer (KLA Tencor Alphastep). The transmission through the dielectric-coated silver films is measured across the visible and infrared spectrum (400 nm to 1800 nm) using a Filmetrics F20 analyzer system, whereas the transmission through the silver-coated membranes is measured across the visible spectrum (400 nm to 750 nm) using a Schott-Fostec DDL fiber optic light source with an Ocean Optics USB4000 spectrometer in a confocal arrangement to accommodate the small aperture of the membranes. Transmission images of the silver-coated membranes under laser illumination at various wavelengths (365 nm, 470 nm, 590 nm) are collected by a Zeiss Axioimager microscope and captured by a monochrome CCD camera.

## Results and Discussion

**Figure 2:**
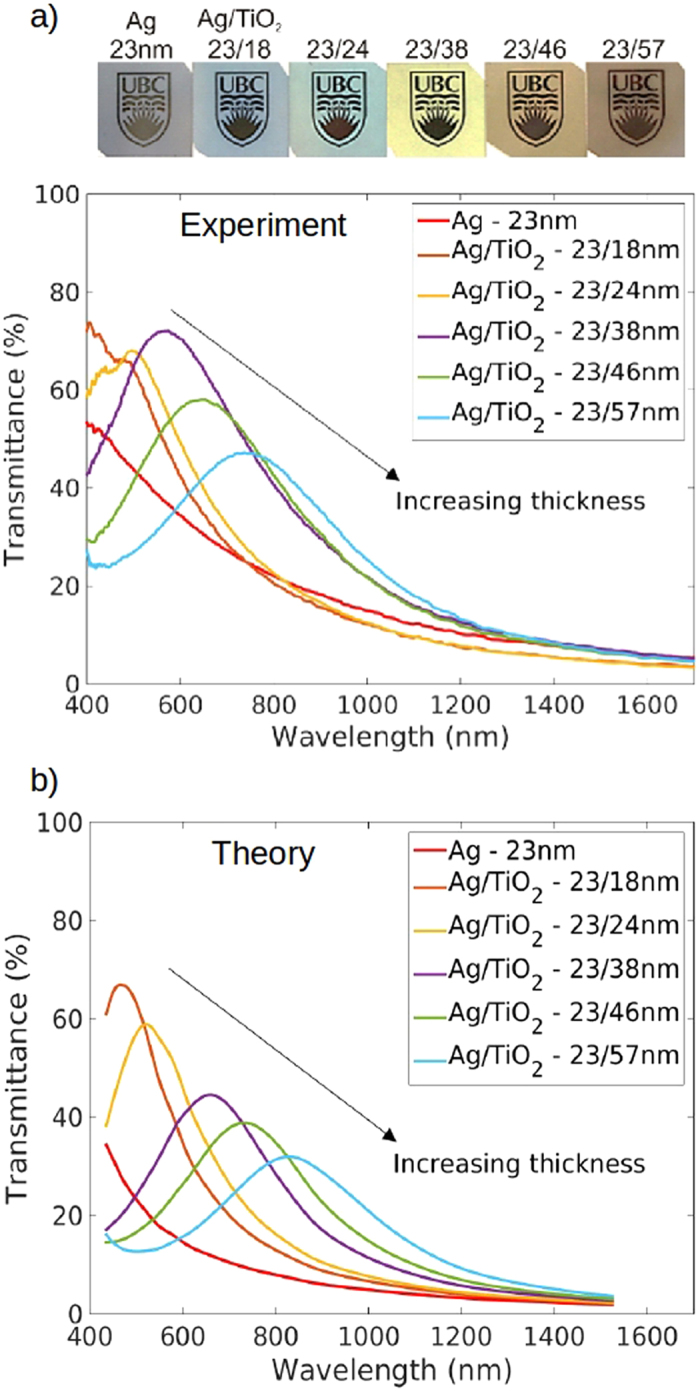
Changing the optical properties of semi-transparent silver by sputtered TiO_2_ coatings. (**a**) Experimental and (**b**) calculated normal-incidence transmittance spectra for 23-nm-thick silver layers that are either uncoated or coated with a TiO_2_ layer ranging in thickness from 18 nm to 57 nm. The experimental spectra are obtained from the average of 5 independent measurements, where each measurement is made from an average of 40 traces. Photographs of the samples placed on the printed UBC logo are shown at the top of panel (**a**) to highlight the visible appearance changes caused by the thin TiO_2_ layer. The leftmost photograph is of uncoated silver and the adjacent images are of coated silver (in order of increasing coating layer thickness from left to right).

Enhanced transmission through silver is most pronounced when it is coated with a high-index, low-loss dielectric, such as TiO_2_. Figure 2(a) shows the measured normal-incidence transmittance spectra of uncoated and TiO_2_-coated silver films, along with photographs of the samples. The transmittance spectra are normalized to free space and expressed as *I*(λ)/*I*_*o*_(λ), where *I*(λ) and *I*_*o*_(λ) are the transmitted spectral intensities through the sample and air, respectively. The TiO_2_ coatings cause the transmittance spectrum of bare silver, which drops monotonically from blue to red, to develop a peak (known as a “transparency band”) at a wavelength dependent on the coating thickness. TiO_2_-coated silver films have transparency bands whose peak wavelength can shift from ~400 nm to ~780 nm by changing the coating thickness from 18 nm to 57 nm. The fabricated samples take on an array of colors including light blue, greenish blue, yellow, and brown. There is good agreement between the trends in the experimental data and theoretical calculations shown in Fig. 2(b). The calculations based on solutions to Maxwell’s equations of the transmission through planar layered media in which the optical constants for silver and TiO_2_ found in ref. 28 and ref. 34, respectively. Slight offset between the peaks of the calculated and measured transparency bands can be reasonably attributed to differences in the assumed optical constants of silver and TiO_2_ and their actual values, surface roughness of the films not described in the model, or a combination of both.

**Figure 3:**
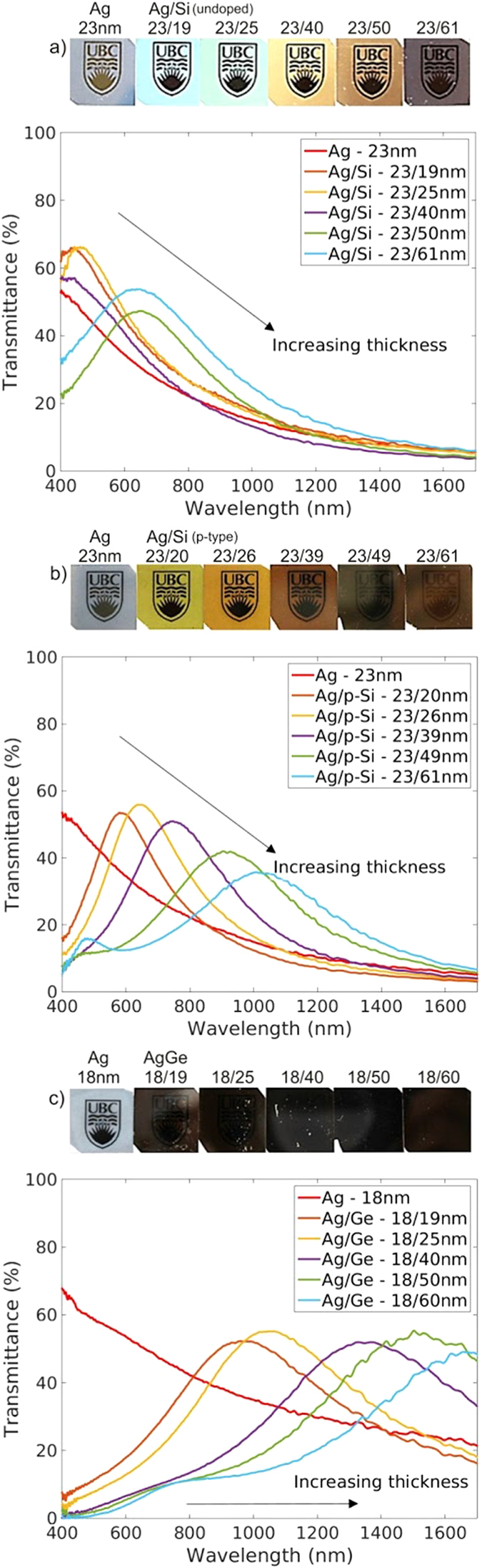
Changing the optical properties of semi-transparent silver by various sputtered elemental semiconductor coatings. Experimental normal-incidence transmittance spectra for (**a**) 23-nm-thick silver coated with sputtered silicon, (**b**) 23-nm-thick silver coated with sputtered p-type silicon, and (**c**) 18-nm-thick silver coated with sputtered germanium. The experimental spectra are obtained from the average of 5 independent measurements, where each measurement is made from an average of 40 traces. Photographs of the samples placed on the printed UBC logo are shown at the top of each corresponding panel to highlight the visible appearance changes caused by the thin layers.

We show that transparency bands can also be induced by coating silver with sputtered semiconductor coatings. Figure 3 shows the measured normal-incidence transmittance of silver films coated with sputtered Si, p-Si, and Ge, along with photographs of the samples. Interestingly, sputtered Si induces visible-frequency transparency bands that are similar in magnitude and spectral position to those induced by sputtered TiO_2_. This suggests that sputtered Si could be used as an alternative to sputtered TiO_2_ in applications such as metal-based heat-reflecting windows or transparent conductors. Coatings made of sputtered p-Si induce transparency bands straddling the red edge of the visible (~600 nm to ~1100 nm), whereas those made of sputtered Ge lie completely outside the visible spectrum (~900 nm to ~1800 nm). Differences in spectra for Si-coated and Ge-coated samples can be explained by large index variations between bulk Si and Ge. Differences in spectra for Si-coated and p-Si-coated samples, on the other hand, are surprising due to the small index variations between bulk Si and p-Si. The observations suggest that the sputtered p-Si coating is compositionally different from the bulk p-Si target from which it originates and requires further investigation. Between the three types of semiconductor coatings used here, induced transparency bands in silver films can be tailored across the entire visible and near-infrared, which may be useful for making absorbers, optical filters, or solar cell coatings. A drawback of using sputtered Si and Ge coatings, however, is that their optical properties are highly dependent on growth conditions^35^,^36^ and not well-characterized, which limits the predictive accuracy of theoretical models.

**Figure 4:**
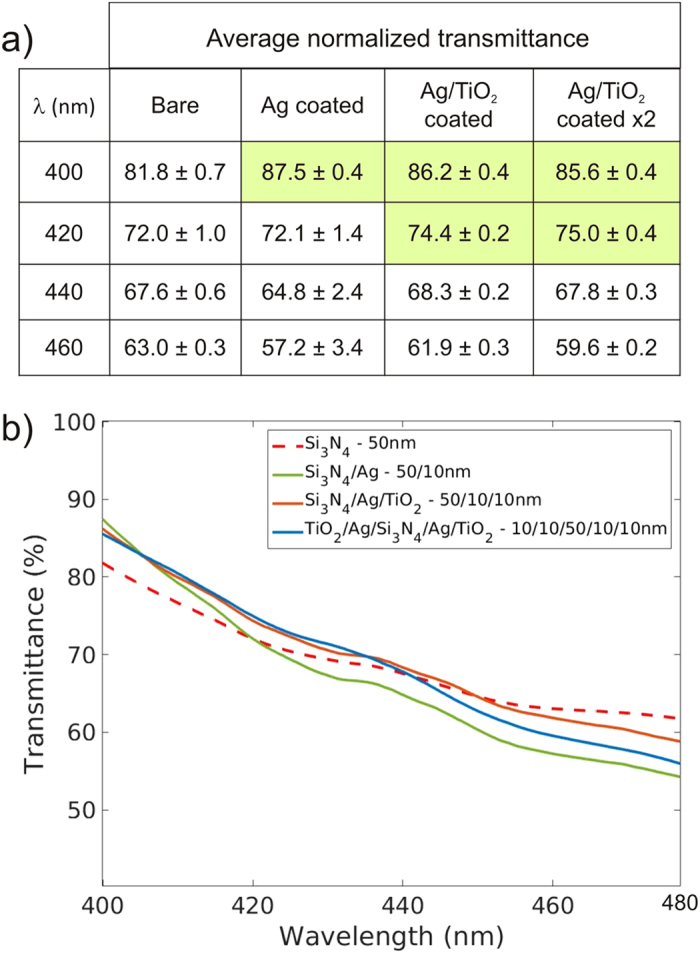
Transmission enhancement of a 50-nm-thick silicon nitride membrane conferred by coating the membrane with 10-nm-thick silver layers in three different configurations: single-sided coating with silver, single-sided coating with silver followed by a 10-nm-thick TiO_2_ passivating layer, and double-sided coating with passivated silver. The transmittance measurements are performed for normal incidence illumination. The average transmittance for each membrane is calculated from 10 independent transmission measurements at different locations on the membrane, where each measurement is made from an average of 150 traces. Baseline bare membrane measurements are obtained using three different samples, to account for slight variations in thickness. (**a**) shows tabulated transmittance values for the bare membrane and the three silver-coated membranes at the wavelengths of 400 nm, 420 nm, 440 nm, and 460 nm. Cells in the table corresponding to transmission enhancement (beyond experiment error) are shaded green. (**b**) shows the average transmittance spectra for the bare membranes (red dashed), the membrane that is coated on a single side by silver (green line), the membrane that is coated on a single side by passivated silver (orange line) and the membrane that is coated on both sides by passivated silver (blue line). The error has a magnitude comparable to the line widths and has not been explicitly plotted for clarity of presentation.

We next show that a 50-nm-thick silicon nitride membrane can be made more transparent by coating with silver. Figure 4(a) compares two sets of average transmittance spectra (in the blue and green parts of the visible spectrum): a control spectra corresponding to a bare membrane and three other spectra corresponding to membranes coated by 10-nm-thick silver layers in different ways — the first by single-sided coating with silver, the second by single-sided coating with silver followed by a 10-nm-thick TiO_2_ passivating layer, and the third by double-sided coating with passivated silver. A 10 nm thickness for the silver layer was chosen because it is the minimum thickness for which sputtered silver forms a continuous film, as inferred from scanning electron microscope images. The spectra are averaged across many measurements to mitigate three sources of uncertainty: the control transmittance spectra are averaged across three bare membranes to account for thickness variations across samples; for any given membrane, the transmittance spectra are averaged over 10 different locations on the membrane to account for local thickness variations; and the transmittance spectrum at each location is averaged over 150 measurements to mitigate random noise from the light source and spectrometer. Compared to the uncoated membrane, all three silver-coated membranes exhibit modest transmission enhancements in the blue part of the visible spectrum beyond the error of the measurements, as highlighted in Fig. 4. The transmission enhancement for all three silver-coated membranes peaks at 400 nm, the lower wavelength bound of the measurement. Coating the membrane on a single side with silver yields the maximum measured enhancement of 6 ± 1%. The passivated silver-coated membranes achieve similar, but slightly smaller, transmission enhancements as the non-passivated one, except over a larger wavelength range.

**Figure 5:**
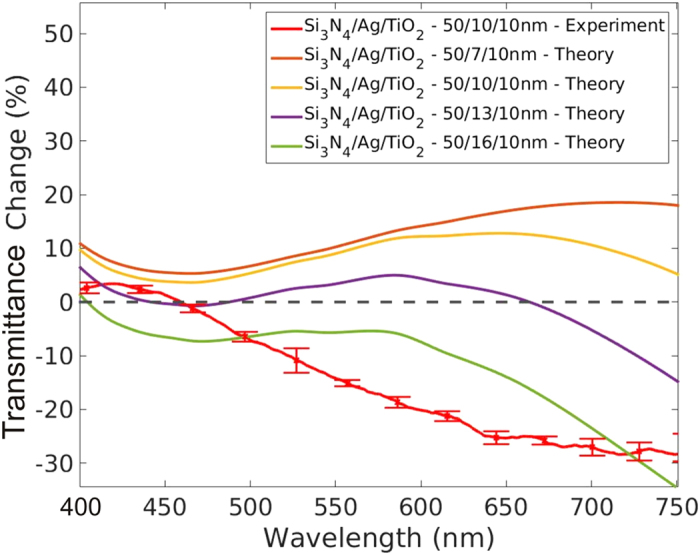
Experimental transmittance change over the entire visible spectrum for a 50-nm-thick silicon nitride membrane coated with a 10-nm-thick silver layer that is passivated by a 10-nm-thick TiO_2_ layer (red line with error bars). Also shown are calculations of the transmittance change for the three-layer system assuming various silver layer thicknesses. The error bars in the experimental measurement represent one standard deviation.

Figure 5 plots the measured transmittance change of the passivated single-side silver-coated membrane over the full visible spectrum, along with the calculated transmittance change for various silver layer thicknesses. The transmittance change is given by [*I*_*c*_(λ) - *I*_*b*_(λ)]/*I*_*b*_(λ), where *I*_*c*_(λ) and *I*_*b*_(λ) are the transmitted spectral intensities through the silver-coated membrane and bare membrane, respectively. The experimental data and theoretical calculations show similar positive transmittance changes at blue wavelengths, but diverge at larger wavelengths. Under ideal conditions of perfect layer planarity and sharp boundaries, the model predicts that very thin silver layers (7 nm) provide greater than 10% transmittance change over the entire visible spectrum. However, increasing the silver layer from 7 nm to 16 nm is sufficient to completely destroy the enhancement effect. The calculated transmission enhancements are predicted to be most robust in the blue part of the spectrum as a function of the silver thickness, which may explain why the measured transmission enhancement is restricted to this frequency range. Experimental enhancement factors will likely improve by making smoother silver layers. The addition of a seed layer of germanium or nickel can produce silver films that are morphologically smoother^37^,^38^, but may also alter the optical constants of silver. This strategy should be explored next.

**Figure 6:**
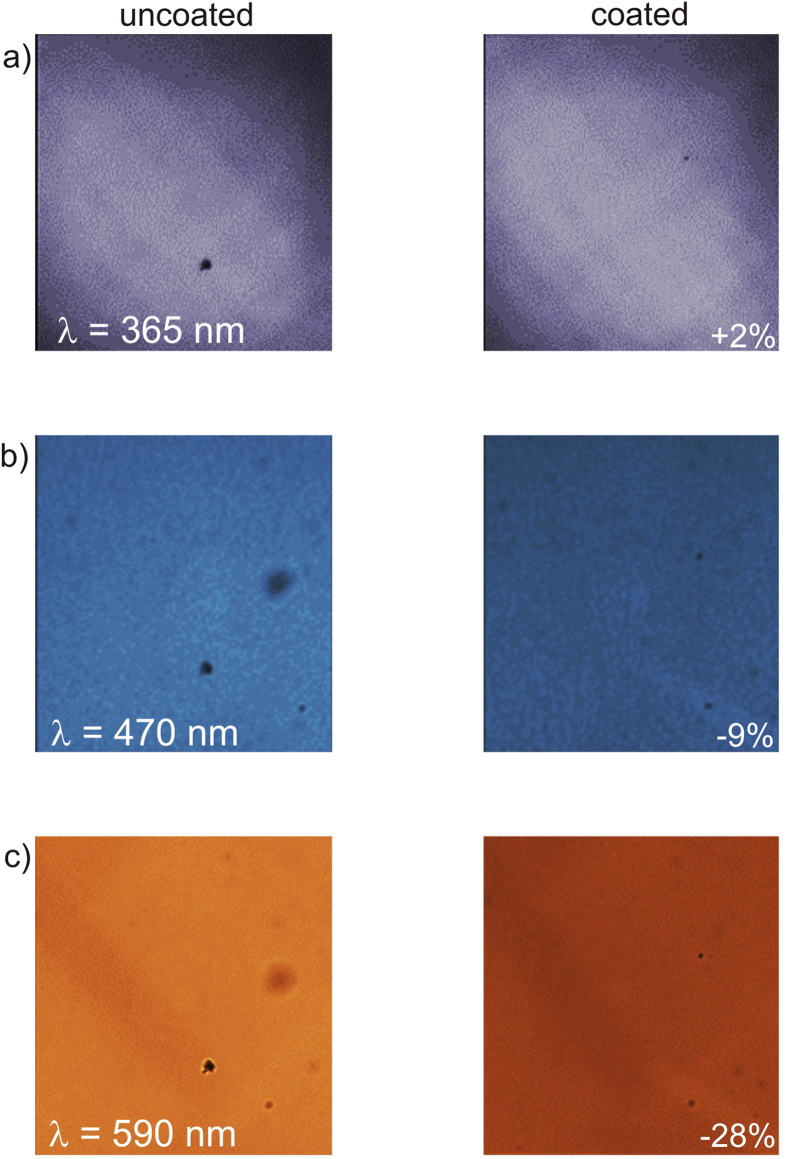
Microscope images of (left column) an uncoated 50-nm-thick Si_3_N_4_ membrane and (right column) an identical membrane coated with 10-nm-thick Ag and 10-nm-thick TiO_2_ under laser illumination at wavelengths of (**a**) 365 nm, (**b**) 470 nm, and (**c**) 590 nm. The images were collected using a monochrome camera and have been false-colored to reflect the color of laser illumination. The percentages on the images in the right column indicate the percent change in the average image brightness relative to the adjacent images in the left column.

To provide further evidence of transmission enhancement conferred by thin silver coatings, Fig. 6 shows comparative microscope images of an uncoated membrane and an identical membrane coated with 10 nm of Ag and 10 nm of TiO_2_ under normal-incidence laser illumination at wavelengths of 365 nm, 470 nm, and 590 nm. The coating causes a modest image brightness increase of about 2% at the wavelength of 365 nm (which is below the 400 nm lower wavelength limit of the spectral measurements in Figs 4 and 5). At wavelengths of 470 nm and 590 nm, the image brightness decreases by 9% and 28%, which are both consistent with spectral measurements in Fig. 5.

## Conclusion

We have proposed through a solution to Maxwell’s equations that the transmission through any optically thin layer can be boosted by adding another thin coating with a susceptibility of opposite sign. This concept has been experimentally and theoretically explored in two configurations. First, we have used subwavelength-thick dielectric layers to increase the transparency of semi-transparent silver films. This effect has been well-studied using metal oxides such as TiO_2_, but has been shown here to also occur using elemental semiconductors such as Si, p-Si, and Ge. Second, we have achieved modest boosts in the transmission through a 50-nm-thick silicon nitride membrane by 10-nm-thick silver coatings in three different configurations — the first by single-sided coating with bare silver, the second by single-sided coating with passivated silver, and the third by double-sided coating with passivated silver. Transmission enhancement of 6 ± 1% over the blue part of the visible spectrum has been achieved. The theoretical limits of transmission enhancements by thin silver layers are generally larger than what has been measured experimentally, but realization of such limits is hindered by the difficulty in fabricating perfectly planar silver films less than 10 nm thick. Future work should explore the use of seeding layers to improve the planarity of the silver layers and the potential existence of the magnetic counterpart of this effect using bi-layers with opposing magnetic susceptibility.

## Additional Information

**How to cite this article**: Shakhs, M. A. *et al*. Boosting the Transparency of Thin Layers by Coatings of Opposing Susceptibility: How Metals Help See Through Dielectrics. *Sci. Rep.* **6**, 20659; doi: 10.1038/srep20659 (2016).
